# Sleep Quality in Shift-Working Nurses: Subjective and Objective Evaluation

**DOI:** 10.3390/ijerph23010064

**Published:** 2025-12-31

**Authors:** Željka Dujmić, Štefica Mikšić, Ivana Barać, Josip Samardžić, Lea Maršić, Petar Samardžić, Zvjezdana Gvozdanović, Ivana Jelinčić, Blaženka Kljajić Bukvić, Marija Barišić, Davorka Čavar-Lovrić, Ružica Mrkonjić, Ivica Mihaljević, Nikolina Farčić

**Affiliations:** 1 Faculty of Dental Medicine and Health Osijek, Josip Juraj Strossmayer University of Osijek, 31000 Osijek, Croatia; zeljka.dujmic@bolnicasb.hr (Ž.D.); smiksic@fdmz.hr (Š.M.); ibarac@fdmz.hr (I.B.); josip.samardzic@gmail.com (J.S.); zvjezdana.gvozdanovic@obnasice.hr (Z.G.); jelincic.ivana@kbco.hr (I.J.); blazenka.bukvic@gmail.com (B.K.B.); marija.barisic@fdmz.hr (M.B.); 2 Faculty of Medicine, Josip Juraj Strossmayer University of Osijek, 31000 Osijek, Croatia; gvozdanovic.lea@gmail.com (L.M.); ivica.mihaljevic@mefos.hr (I.M.); 3 General Hospital Dr. Josip Benčević Slavonski Brod, 35000 Slavonski Brod, Croatia; petar.samardzic501@gmail.com (P.S.); dacavar@gmail.com (D.Č.-L.); 4 General County Hospital Našice, 31500 Našice, Croatia; 5 University of Applied Sciences in Bjelovar, 43000 Bjelovar, Croatia; 6 University Hospital Centre Osijek, 31000 Osijek, Croatia; 7 University Hospital Dubrava, 10000 Zagreb, Croatia; ruzicam@kbd.hr

**Keywords:** sleep quality, shift work, nurses, PSQI, FitBit Charge 3

## Abstract

**Highlights:**

**Public health relevance—How does this work relate to a public health issue?**

- Highlights the growing burden of sleep disturbances among nurses, a workforce essential for maintaining patient safety and healthcare quality.
- Addresses the impact of shift work on circadian health, an important determinant of long-term wellbeing and occupational functioning.

**Public health significance—Why is this work of significance to public health?**

- Demonstrates a clear discrepancy between subjective and objectively measured sleep quality, underscoring the need for reliable assessment tools in occupational health.
- Provides evidence that shift work is associated with objectively poorer sleep, supporting public health efforts aimed at improving working conditions for healthcare workers.

**Public health implications—What are the key implications or messages for practitioners, policy makers and/or researchers in public health?**

- Calls for targeted interventions and organizational changes to improve sleep health among shift-working nurses, which may enhance performance and reduce fatigue-related errors.
- Emphasizes the importance of integrating wearable technology into routine occupational health monitoring to better detect and address sleep-related risks.

**Abstract:**

Background: It is well-known that rotating shift work disrupts the circadian rhythm and sleep quality in nurses. With this study, we aimed to compare subjectively and objectively measured sleep quality in nurses, specifically focusing on any differences that arose in relation to shift work. Methods: This prospective, observational, cross-sectional study was conducted in 2025 in Croatia; a total of 140 nurses participated. Sleep was assessed using the Pittsburgh Sleep Quality Index (PSQI) and FitBit Charge 3 smartwatch, which monitored sleep over an eight-day period. Results: Most nurses rated their sleep as good or very good, but according to the PSQI questionnaire, all participants were classified as poor sleepers (PSQI > 5). Objective smartwatch measurements showed that nurses working only day shifts had higher sleep scores (median 77, IQR 75–80 vs. 73, IQR 68–76; *p* < 0.001), significantly longer total sleep duration (median 6.4, IQR 6.3–7.1 vs. 5.5, IQR 5.2–6.2 h; *p* < 0.001), and longer durations of all sleep stages compared with those working rotating shifts. Conclusions: Most nurses subjectively rated their sleep as good or very good; however, according to the PSQI questionnaire results, all were classified as poor sleepers, with no significant difference related to shift work. Objective measurements using the smartwatch indicated that rotating shift work is associated with significantly poorer sleep quality.

## 1. Introduction

Sleep quality represents an individual’s subjective satisfaction with various aspects of the sleep experience, and it is recognized as an essential component of overall health and well-being. Adequate sleep contributes to optimal physical, psychological, and functional health, thereby enhancing quality of life [1,2]. Conversely, chronic sleep deprivation has been found to be associated with a wide range of adverse health outcomes, including metabolic and cardiovascular diseases, inflammation, depression, anxiety, and cognitive dysfunction, and it may even increase overall mortality risk [3,4]. Furthermore, longitudinal studies indicate that improvements in sleep duration and quality can lead to measurable enhancements in individuals’ quality of life [5].

Shift work is an integral and indispensable part of the healthcare system because it ensures continuous care for patients 24 h a day, 7 days a week. Although rotating shift work is necessary, it brings about numerous challenges for physiological and psychological processes, especially the circadian rhythm, which regulates the cycle of sleep and wakefulness [6,7]. Working at night during the period when the organism is biologically programmed to rest leads to circadian desynchrony. The consequences of this are disturbances to the internal clock, acute lack of sleep, frequent sleep disorders, and chronic fatigue and exhaustion [6,7,8]. Numerous studies have shown that nurses who work night shifts experience significantly poorer sleep quality compared with those who work exclusively during the day [9,10]. According to recent studies, as many as 60% of nurses who work at night report poor sleep quality [9]. Such results are concerning because poor sleep quality increases the risk of medical errors and reduces concentration and overall work efficiency [10]. It was observed that when working night shifts, there is a 30% higher risk of work-related errors compared with working day shifts [11]. Nurses who work at night often report symptoms of insomnia and severe fatigue, which further compromises their alertness and productivity [10,11,12] and increases the risk of accidents and injuries at work [13]. Previous research has shown that most night shift nurses prefer to sleep at night during their days off. Among the identified sleep adjustment patterns, the nap proxy and sleep strategies were found to be associated with poorer adaptation to night shift work, including greater sleep disturbances and increased cardiovascular risks. These findings suggest that maladaptive coping strategies may exacerbate the negative health consequences of night shift schedules and highlight the importance of promoting healthier sleep–wake adjustment behaviors among nurses [14].

In the assessment of sleep quality, two main methodological groups of instruments are used: subjective and objective methods [15]. Subjective methods are based on self- assessment and include standardized questionnaires, sleep diaries, and interviews, which provide insight into individuals’ perceptions of their sleep experience. Self-perception of sleep quality is challenging because no universally accepted gold standard exists. One of the most commonly used approaches is the application of standardized questionnaires, among which the Pittsburgh Sleep Quality Index (PSQI) stands out. The PSQI was developed in 1988 to provide a reliable and valid measure of sleep quality and distinguish between “good” and “poor” sleepers, which is practical for both subjects and clinicians [16].

Objective methods rely on measurement devices and laboratory procedures and assume that subjective judgement has minimal influence. Polysomnography [PSG] is the gold standard in sleep assessment because it allows for the most precise analysis of its structure, including non-rapid eye movement (NREM) and rapid eye movement (REM) sleep phases, and is based on the simultaneous recording of multiple physiological parameters. Although it provides very detailed data, PSG requires specialized laboratory conditions and is associated with high costs, which limits its application in long-term and field studies [17].

Actigraphy is a non-invasive method used to objectively assess sleep–wake patterns. It is based on the use of a fan accelerometer that records a person’s body movements. By analyzing the rhythm and frequency of movements, it is possible to determine sleep duration and efficiency, its fragmentation, and the basic patterns of the circadian rhythm. Unlike PSG, actigraphy allows for the long-term monitoring of sleep in natural conditions, which has been proven to be practical in epidemiological and longitudinal studies. However, its precision is lower compared with PSG, particularly in distinguishing between different sleep stages and detecting brief awakenings [18,19].

Technological advances have enabled the development of commercial wearable devices, such as the Fitbit, Oura Ring, Garmin, or Apple Watch, which combine accelerometry with additional physiological signals, including heart rate, heart rate variability, oxygen saturation, and peripheral temperature. These devices have demonstrated satisfactory reliability in assessing total sleep duration, while their accuracy in differentiating sleep stages and detecting awakenings remains limited [20,21].

To date, no study in Croatia has compared subjective and objective assessments of sleep quality in nurses in the context of shift work. This study aims to fill the existing gap in the literature and contribute to our understanding of sleep quality in the nursing population. The aim of this study was to examine the differences in subjectively and objectively measured sleep quality in nurses in relation to shift work.

## 2. Materials and Methods

### 2.1. Study Design and Participants

**Figure 1:**
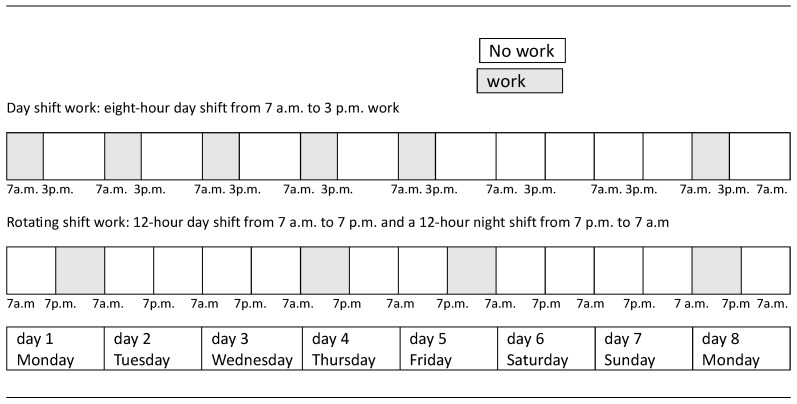
Nurses’ work schedule during the measurement period in relation to shift type.

We performed this prospective, cross-sectional, observational study at Dr. Josip Benčević General Hospital in Slavonski Brod, Croatia, from January to July 2025. The study included 140 nurses of all educational levels divided into 2 groups: those who work rotating shifts and those who work day shifts. The sleep duration of the nurses was monitored for eight consecutive days, during which the participants used a wearable smart device to objectively record their sleep parameters. At the same time, subjective data on sleep quality were collected via a standardized questionnaire. During the study, shift protocols were applied that included a limited number of consecutive night shifts, ensuring at least two days of rest after the night shift. The group that works day shifts work only the day shift from 7 a.m. to 3 p.m., Monday through Friday, with two days off on Saturday and Sunday. The group that works shifts followed a rotating schedule, consisting of a 12 h day shift from 7 a.m. to 7 p.m. and a 12 h night shift from 7 p.m. to 7 a.m., followed by two days off (Figure 1). Nurses included in the study worked exclusively in their assigned shift schedule (day shifts or rotating shifts) since employment or for at least two years prior to study inclusion, ensuring stable and long-term exposure to the respective type of shift work.

Inclusion criteria: Nurses employed in clinical departments who are in direct contact with patients, with at least two years of work experience, permanent employment status, and voluntary consent to participate.

Exclusion criteria: Diagnosed sleep disorders and regular use of sleeping medications. Participants with a self-reported history of psychiatric disorders (including mood or anxiety disorders and depression) or those using psychotropic medications known to affect sleep were excluded from the study.

The structured questionnaire with demographic data contained questions about age, gender, years of works experience, education, shift work, body weight and height. In addition, information on diagnosed sleep disorders and current therapy use was collected to assess eligibility and apply the exclusion criteria.

Statistical methods to calculate the appropriate number of respondents were applied as follows: to detect a medium effect (d = 0.5) in the difference in sleep duration between two independent groups, with a significance level of 0.05 and a power of 0.80, the minimum required sample size was 128 subjects (G*Power, ver. 3.1.9.4). Expected values for sleep duration were based on previously published data in nurses working different shift schedules [22], where sleep duration was reported as a key outcome variable across shift types.

### 2.2. Sleep Quality Questionnaire—Pittsburgh Sleep Quality Index (PSQI)

The Pittsburgh Sleep Quality Index [16] is a standardized psychometric instrument used to self-assess one’s subjective sleep quality over the previous month. The questionnaire consists of 24 items, of which 19 are completed by the respondent, while the remaining 5 are related to the respondent’s bed partner. Based on the responses, seven components are formed, which together form the overall assessment of sleep quality: subjective sleep quality, sleep efficiency (the ratio between time spent in bed and actual sleep time), sleep duration, sleep latency, sleep disturbances (e.g., awakenings, pain, difficulty breathing), use of sleep medications, and daytime dysfunction (concentration problems, sleepiness, reduced functionality) [16,23,24]. The sum of the seven components provides the global PSQI score, which ranges from 0 to 21, with higher scores indicating a worse subjective assessment of sleep quality [16]. A total score greater than 5 indicates poorer sleep quality, with higher scores associated with greater intensity of sleep disturbance in fewer categories or with moderate intensity of sleep disturbance in several different categories. The internal reliability of the entire scale, expressed as Cronbach’s alpha, is 0.80, indicating that the questionnaire is a good tool for assessing sleep quality in this sample. The Croatian translation of the PSQI was used [25]. The PSQI is free to use for educational and noncommercial research purposes [16]. The validity of the PSQI has been confirmed by several studies in different languages [26,27,28] and in different patient populations [29,30].

### 2.3. Sleep Monitoring Using the Fitbit Charge 3 Smartwatch

The Fitbit Charge 3 smartwatch [21,31,32,33,34,35] was used in this study. It is an advanced wearable fitness device that has the ability to monitor throughout the day and the entire week 24/7. It tracks heart rate, exercise, oxygenation, all stages of sleep, number of steps, distance, active minutes, calorie consumption, and targeted exercise mode [21,31,32,33,34,35]. Data can be stored in PDF or Excel format by connecting the device to the desktop application. The smartwatch assesses overall sleep quality daily and weekly by summing individual sleep duration and sleep quality scores. Sleep duration includes time spent in sleep and wakefulness, i.e., how long a dream lasts. The longer the dream lasts, the better the score. Sleep score includes the duration of deep sleep and REM sleep. The longer the periods of deep sleep and REM sleep, the better the score. Recovery includes sleep pulse rate and relaxation, with accelerated pulse rate and restless sleep lowering the overall sleep score. All sleep events (sleep bouts) automatically detected by the Fitbit Charge 3, including both nighttime and daytime sleep, were combined and included in the analysis. Naps were included and contributed to total sleep duration and overall sleep quality scores as calculated by the device algorithm. Sleep quality was also categorized using the Fitbit sleep score (0–100). Based on Fitbit’s general cut-offs, scores were classified as follows: <60 = poor, 60–79 = moderate, 80–89 = good, and 90–100 = excellent sleep quality.

According to recent validation studies, the Fitbit Charge Series 3 and 4 show moderate-to-high accuracy when it comes to estimating total sleep duration (accuracy around 85–88%), while the precision in distinguishing between sleep and wakefulness remains limited [33,34,35]. The device shows results comparable to actigraphy but underestimates deep sleep and overestimates light sleep compared with polysomnography [20,33,34].

For this study, 15 Fitbit Charge 3 smartwatches were used. Participants were instructed to wear the Fitbit Charge 3 smartwatch continuously on their non-dominant wrist for eight consecutive days and nights, including while sleeping. The device was removed only, when necessary, such as bathing, showering, swimming, or other activities involving prolonged exposure to water. Participants were instructed to charge the device once daily, preferably during periods when the watch was removed (e.g., while showering), to minimize data loss. After charging, participants were instructed to immediately re-apply the device, ensuring a secure fit to allow for accurate sleep and activity tracking throughout the measurement period.

### 2.4. Ethical Considerations

This study was approved by the Ethics Committee of the Dr. Josip Benčević General Hospital Slavonski Brod (protocol code of approval 040000/24-37, approval date: 10 June 2024). All participants were informed about the objectives of this study and voluntarily signed an informed consent form. The participants’ anonymity was guaranteed.

### 2.5. Data Analysis

Categorical data are presented as absolute and relative frequencies. Differences in categorical variables were tested using Fisher’s exact test. The normality of the distribution of numerical variables was tested using the Shapiro–Wilk test. Continuous data are described by the median and the limits of the interquartile range. The Mann–Whitney U test was used to assess the differences in continuous variables regarding whether or not the participants work shifts (Hodges–Lehmann median difference and 95% CI shown) [36]. All *p*-values are two-sided. The significance level was set at Alpha = 0.05. The statistical program MedCalc^®^ Statistical Software version 23.3.4 (MedCale Software Ltd., Ostend, Belgium; https://www.medcalc.org, accessed on 25 August 2025) was used for the data analysis.

## 3. Results

This study included 140 respondents, of whom 25 (17.9%) were men and 115 (82.1%) were women (Table 1). The median age of the respondents was 44 years, ranging from 20 to 64 years. A total of 67 (47.9%) respondents had a secondary education, and according to length of service, the least number of respondents, 12, had 6–10 years of service (8.6%). In total, 70 (50%) respondents worked shifts.

Nurses working rotating shifts and those working day shifts accumulated an equal total number of working hours during the measurement period (48 h), despite differences in shift length and number of working days. The distribution of years of nursing experience was comparable between the two groups (Table 2).

The respondents who worked rotating shifts were significantly younger than those who did not (median 40 years vs. 45 years) (Mann–Whitney U test, *p* = 0.04), while there were no significant differences in body mass index based on type of work (Table 3).

**Figure 2:**
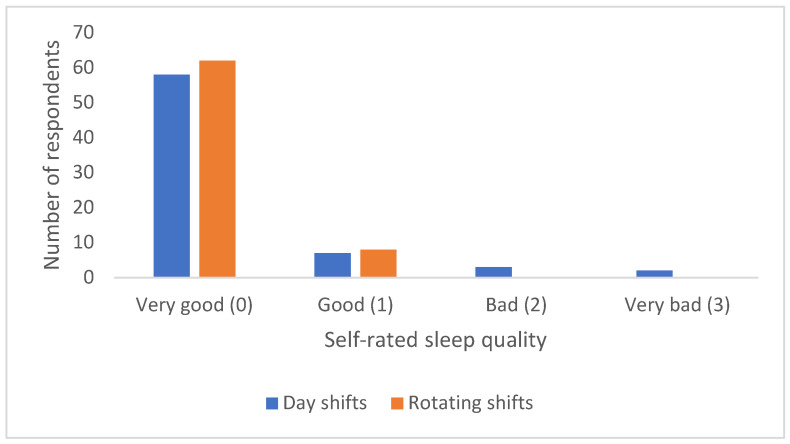
Sleep assessment in relation to type of shift work.

Subjective sleep quality was determined by answering the question of how the respondents would rate their sleep over the last month (Figure 2). The majority of respondents reported very good sleep over the past month 120 (85.7%), with few reporting bad 3 (2.1%) or very bad sleep 2 (1.4%). There was no significant difference in subjective sleep quality with respect to type of shift work (Fisher’s exact test, *p* = 0.17).

There were no significant differences in sleep latency in relation to type of shift work (Table 4). A total of 29 (21%) of the respondents slept for more than seven hours a night, and 19 (14%) slept for less than five hours, with no significant difference in relation to shift work (χ^2^ test, *p* = 0.16). It was observed that shift work significantly impairs sleep efficiency (Fisher’s exact test, *p* = 0.002). Most respondents, 85 (61%) of them, reported 1–9 problems that caused them to wake up during the night or early in the morning, and only 1 (1%) respondent stated that they had no sleep disorders. There were no significant differences in the distribution of respondents according to sleep disturbance in relation to shift work (Fisher’s exact test, *p* = 0.78). There were no significant differences in the use of sleep medications (Fisher’s exact test, *p* = 0.50) and daytime sleepiness in relation to shift work (Fisher’s exact test, *p* > 0.99) (Table 4).

The median subjective sleep quality was 0 (very good), ranging from 0 (very good) to 3 (very bad) (Table 5). There were no significant differences in the subjective sleep quality score in relation to shift work (Mann–Whitney U test, *p* = 0.27). Likewise, there were no significant differences in subjective sleep quality scores and in the total sleep scale scores in relation to shift work. Considering the total sleep scale values, all respondents are poor sleepers (score of 5 and above).

Respondents who work day shifts have significantly better sleep parameters compared with those who work rotating shifts (Table 6): they have significantly longer total sleep (median 6.4 vs. 5.5 h) (Mann–Whitney U test, *p* < 0.001), have a higher sleep score (77 vs. 73) (Mann–Whitney U test, *p* < 0.001), and have a longer duration of all sleep stages, including deep sleep (Mann–Whitney U test, *p* = 0.01), light sleep (Mann–Whitney U test, *p* < 0.001), and REM sleep phase (Mann–Whitney U test, *p* < 0.001).

Responders who worked rotating shifts took a significantly higher number of steps per day (median: 10,662 vs. 7295) (Mann–Whitney U test, *p* < 0.001) (Table 6).

Based on the sleep score results collected using the smartwatch, the majority of respondents, 104 (74.3%), had a moderate sleep score (Table 7). With regard to the sleep score values, it was observed that shift work was associated with a worse quality of sleep (Fisher’s exact test, *p* < 0.001) objectively measured with the smartwatch.

## 4. Discussion

The results of this study indicate an interesting and significant discrepancy between respondents’ self-assessment of sleep quality, the standardized PSQI questionnaire, and objective data collected from a smartwatch (wearable technology). Three different data sources, namely self-assessment, PSQI, and the Fitbit Charge 3 smartwatch, led to different results.

Specifically, the vast majority of respondents rated their sleep as good or very good (96.4%), but according to the PSQI, 100% of respondents were classified as poor sleepers (PSQI > 5). No statistically significant difference was found between nurses who work rotating shifts and those who work exclusively in the daytime, neither according to the respondents’ self-assessment nor according to the PSQI questionnaire, but it was observed that shift work significantly impairs the sleep efficiency of nurses who work rotating shifts.

These results are unexpected since the results of most studies suggest that shift work generally leads to poorer subjective sleep quality in nurses who work rotating shifts, which is in contrast to the results of this study [37,38,39,40,41,42]. Flo et al. reported that rotating shift work significantly increased respondents’ ratings of poor sleep quality compared with those who engaged in fixed daytime work [37]. In a study of 510 healthcare workers in Saudi Arabia, the results indicated that healthcare workers working rotating shifts had significantly higher mean PSQI scores compared with those working only daytime shifts [38]. A study in Spain involving 635 nurses confirmed this pattern, reporting a mean PSQI score of 6.8, with significant differences between groups: nurses who worked night shifts had poorer sleep quality than those who worked daytime shifts [39]. A cross-sectional study of 513 nurses found that rotating shift work was significantly associated with poor sleep quality [40]. A study of 711 nurses in China found out that 90.1% of those working night shifts reported poor sleep quality, with work experience, education, quality of sleep compensation, and daily routine being the key factors associated with poorer sleep [41]. A meta-analysis conducted in China found that 39.2% of Chinese healthcare workers have poor sleep quality. Higher rates of sleep disorders are associated with female gender, lower PSQI scores, nursing occupation, and rotating shift work [42]. One possible explanation for this finding in this study is that a simple sleep questionnaire likely captures a general impression rather than the actual duration and structure of sleep. Healthcare workers may underestimate sleep problems; they may be trying to maintain an image of professional competence, or perhaps prolonged fatigue is becoming the “new normal”.

Self-reported sleep quality showed a very optimistic picture, with 96.4% of respondents rating their sleep as good or very good. We believe that these findings suggest a positive bias and adaptation to chronically disrupted sleep patterns. It seems that nurses, due to their long-term demanding environment, have developed cognitive and emotional coping mechanisms that enable them to perceive their sleep as satisfactory, even though objective data suggest otherwise. Nurses working night shifts often adopt various coping strategies to mitigate the negative effects of rotating shift work on sleep quality. The most common strategies include creating favorable sleep conditions and behaviors, balancing rest and activity time, accepting irregular sleep patterns, and seeking social support [43]. These findings are consistent with previous research that indicated that rotating shift work significantly impairs sleep efficiency, as measured by the ratio of hours of sleep to time spent in bed. A likely explanation is that nurses working rotating shifts create favorable sleep conditions and behaviors and spend more time in bed trying to fall asleep due to irregular sleep patterns. In addition to these individual factors, shift work organization is also important. When a hospital implements favorable shift protocols, such as a limited number of consecutive night shifts and ensuring at least two days of rest after a night shift, circadian rhythm disturbances are milder and subjective perceptions of sleep quality are more favorable. Such a work organization was present in this study. Research from around the globe emphasizes that at least two consecutive days of rest are required for full recovery after working a night shift, and at least three days of rest are required after working two consecutive night shifts [44,45].

One possible explanation is that nurses who work day shifts also experience high levels of occupational stress, which in itself can negatively affect sleep quality. It is therefore possible that professional workload reduces differences between groups when subjective questionnaires are used, which can explain the high levels of stress and workload present in the nursing profession in general, regardless of the work regime [46,47]. Previous studies have also shown that nurses who work exclusively during the day may also have poor sleep quality, which is often associated with burnout [48].

An additional explanation may be found in the so-called “healthy worker effect” [49]. For instance, nurses who remain in rotating shift work for a long time represent a more resilient group, while those who find it more difficult to tolerate night shifts more often switch to day work or leave more demanding jobs. Also, the respondents who worked rotating shifts in this study were more active, taking significantly more steps per day. Such a selection may lead to an apparent equalization of subjective perceptions of sleep quality between groups, although objective measurements still show reduced sleep duration, particularly in terms of deep and REM sleep, which do not return to levels comparable to those of day-shift workers. Similar results have been reported by Huang et al., who found that the prevalence of poor sleep quality remains high, even among nurses who have stopped working night shifts (about 56%), while among those who continue to work at night, it is 62% [50]. A recent meta-analysis of studies published between 2000 and 2020 that assessed sleep quality using the Pittsburgh Sleep Quality Index (PSQI) reported significantly poorer sleep quality among nurses working rotating and fixed night shifts compared with those working fixed day shifts. These findings further support the robustness of PSQI as a widely used instrument for evaluating sleep disturbances in shift-working nursing populations [51].

In contrast to subjective measures, the objective sleep quality parameters used in this study clearly demonstrated the negative impact of shift work. Nurses working rotating shifts slept for about one hour less overall (5.5 h) than those who worked exclusively during the day (6.4 h), and all sleep stages were reported to be shortened in nurses working rotating shifts. Accordingly, their average sleep score was statistically significantly lower.

These results are consistent with the findings of most previous studies that have used objective wearable sleep measurement devices. Hafycz et al. used Fitbit smartwatches and found that after night shifts, subjects had the poorest sleep quality, with the least total, light, deep, and REM sleep compared with those who worked other shifts [52]. Developing upon Hafycz et al.’s study, in this study, nurses working rotating shifts also showed poor sleep quality, with even shorter durations of all sleep stages. Our findings are consistent with an Italian study conducted with a sample of 37 nurses who wore an actigraph device for five days to assess their daytime activity levels, nighttime sleep parameters, and daytime rest. In the aforementioned study, it was found that night shifts significantly impaired sleep duration compared with day shifts [53]. Similarly, a Canadian study by Korsiak et al. that included a large sample of 294 hospital workers who wore an actigraph device for one week showed that rotating shift work resulted in shorter and poorer sleep, especially after working night shifts, where shift workers slept for an average of 20–30 min less [54]. In our study, however, the difference was about one hour, indicating a more serious impact. The results of this study are consistent with a study of 64 hospital nurses, half of whom worked rotating shifts, whose sleep duration was measured over six consecutive days using a Fitbit Charge 3. Nurses working rotating day and night shifts had significantly shorter total sleep duration, longer sleep latency, and lower sleep efficiency compared with those working day shifts. Particularly unfavorable values were observed in nurses working three or more consecutive night shifts, confirming that the frequency and duration of night shifts further worsen sleep quality [55]. Unlike previous studies reporting poor subjective sleep quality among nurses, most participants in the present study rated their sleep as good or very good. This may be attributed to the typical shift schedule in our hospital, in which a single night shift is followed by two days off, potentially supporting better perceived sleep quality. A Polish study of 126 nurses reported that prolonged night and rotating shift work leads to circadian rhythm disruption, reduced sleep quality, and cumulative negative consequences for nurses [56].

More recent research has further expanded our understanding of the effect of night work by including the partners of healthcare workers. For example, a 2025 Japanese study involving 30 nurses and their partners who wore Fitbit devices for 14 days found that night work was significantly associated with poorer sleep quality. In addition, the presence of night shifts among nurses also disrupted the sleep of their spouses [57]. This shows that the negative effects of shift work go beyond the workers themselves to the family environment. In this context, rotating shift work and night work have multiple consequences for the physical, psychological, and social health of nurses. Given that the effects of shift work go beyond the individual and affect the family and social and organizational dynamics [58], it is necessary to design interventions that will address all these levels.

### 4.1. Implications for Practice, Education, and Future Research

The findings of this study indicate the need for the systematic monitoring of sleep quality in nurses working rotating shifts using a combination of subjective and objective assessment methods. Incorporating wearable devices into regular health assessments may contribute to the earlier identification of risks associated with chronic sleep deprivation and reduce the negative impact on patient safety and work efficiency.

In nursing education, it is recommended that greater emphasis be placed on sleep hygiene, strategies for adapting to shift work, and recognition of discrepancies between subjective and actual sleep quality. Healthcare institutions should encourage an organizational culture that supports employee health through the optimization of shift schedules, provision of rest time, and educational programs focused on maintaining health and professional skills.

Future research should focus on performing a more detailed examination of the long-term effect of rotating shift work on the physical, mental, and social health of nurses using a combination of subjective and objective sleep assessment methods. Future studies could examine daytime and nighttime sleep separately to provide a more detailed understanding of sleep patterns in shift-working nurses. Also, future research should include a larger number of subjects and additionally take into account lifestyle habits and factors such as diet, physical activity levels, and stress, which can have significant impacts on sleep quality. The use of a longitudinal study design is recommended to generate a better understanding of why healthcare professionals often underestimate their own sleep and to develop more effective measures to preserve their health and work ability.

### 4.2. Research Limitations

This study has several important limitations that should be considered when interpreting the results. This study was performed with a relatively small sample of nurses from a single healthcare facility, which limits the generalizability of the findings to the wider population. It should also be noted that Fitbit devices are practical for monitoring sleep in everyday settings, but they do not reach the precision of polysomnography, especially when it comes to distinguishing individual sleep stages and detecting brief awakenings. Objective sleep monitoring was only conducted for eight days, which is not long enough to provide detailed insight into long-term sleep patterns or changes that may occur during different types of shifts. This study did not separate daytime and nighttime sleep in the analysis, which may limit comparisons with studies reporting sleep by shift type and this should be addressed in future research. Also, sleep efficiency could not be assessed due to the unavailability of time in bed data in the exported smartwatch dataset; future studies should ensure the collection of these data. Subjective assessment of sleep quality may have also been influenced by professional stress, adjustment to chronic fatigue, or the need to respond in a socially desirable manner. This study did not systematically collect data on lifestyle factors such as diet, caffeine intake, physical activity, or family obligations, all things which may also affect sleep patterns.

## 5. Conclusions

The results of this study show that there is no significant difference in the subjective assessment of sleep quality between nurses who work rotating shifts and those who only work day shifts. However, objective measurements using the Fitbit wearable device (Fitbit, Inc., San Francisco, CA, USA) show that rotating shift work is associated with poorer sleep quality. Nurses who worked rotating shifts slept less and had a lower total sleep score and a shorter duration of deep and REM sleep compared with respondents who work day shifts.

The obtained results indicate that assessing sleep in a subjective manner is not always a reliable indicator of real sleep quality. By combining the PSQI questionnaire and wearable devices that record the physiological indicators of sleep, a more realistic and complete picture of sleep habits and actual rest can be achieved.

## Figures and Tables

**Table 1:** Respondents according to basic characteristics.

Gender [*n* (%)]	
Male	25 (17.9)
Female	115 (82.1)
Age (years) [Median (IQR)]	44 (34–51)
Level of education [*n* (%)]	
Secondary nursing school	67 (47.9)
Undergraduate nursing degree	38 (27.1)
Graduate nursing degree	35 (25.0)
Length of work experience [*n* (%)]	
0–5 years	30 (21.4)
6–10 years	12 (8.6)
11–20 years	31 (22.1)
21–30 years	33 (23.6)
More than 30 years	34 (24.3)
Rotating shift work [*n* (%)]	
No	70 (50)
Yes	70 (50)

*n*—number of respondents; IQR—interquartile range.

**Table 2:** Number of sleep sessions (sleep bouts), total hours worked, number of working days during the measurement period, and years of nursing experience in relation to type of shift work.

Variable	Nurses Working Rotating Shifts	Nurses Working Day Shifts
Number of sleep sessions (bouts of sleep)	8 sleep sessions	8 sleep sessions
Number of hours worked (labor hours)	12 h × 4 shifts = 48 h	8 h × 6 shifts = 48 h
Days of work	4 days	6 days
Years of nursing experience [*n* (%)]		
0–5 years	15 (21.4)	15 (21.4)
6–10 years	8 (11.4)	4 (5.7)
11–20 years	14 (20)	17 (24.2)
21–30 years	16 (22.8)	17 (24.2)
More than 30 years	17 (24.2)	17 (24.2)

*n*—number of respondents.

**Table 3:** Age and body mass index (BMI) of respondents in relation to type of shift work.

	Median (Interquartile Range)		Difference	95 % Confidence Interval	*p* *
	Nurses Working Day Shifts	Nurses Working Rotating Shifts			
Age (years)	45 (38–51)	40 (26–48)	−4	−10 to 0	0.04
Body mass index (kg/m^2^)	25.51 (23.72–301.0)	24.81 (22.47–27.68)	−0.85	−2.26 to 0.48	0.21

* Mann–Whitney U test (Hodges–Lehmann median difference).

**Table 4:** Assessment of sleep quality using the PSQI questionnaire (sleep latency, sleep duration, sleep efficiency, sleep disturbance, use of sleep medication, and daytime sleepiness) in relation to type of shift work.

Domains PSQI Questionnaire		Number (%) of Respondents			*p*
		Nurses Working Day Shifts	Nurses Working Rotating Shifts	Total	
Unable to fall asleep within 30 min/latency	Less than once a week	48 (69)	40 (57)	88 (63)	0.16 *
	Once or twice a week	22 (31)	30 (43)	52 (37)	
Sleep duration	>7 h	11 (16)	18 (26)	29 (21)	0.22 *
	6–7 h	32 (46)	21 (30)	53 (38)	
	5–6 h	19 (27)	20 (29)	39 (28)	
	<5 h	8 (11)	11 (16)	19 (14)	
Sleep efficiency	≥85%	58 (83)	44 (63)	102 (73)	0.002 **
	75–84%	9 (13)	18 (26)	27 (19)	
	65–74%	0	7 (10)	7 (5)	
	<65%	3 (4)	1 (1)	4 (3)	
Sleep disturbance/frequency of problems	0	1 (1)	0	1 (1)	0.78 **
	1–9	44 (63)	41 (59)	85 (61)	
	10–18	23 (33)	26 (37)	49 (35)	
	19–27	2 (3)	3 (4)	5 (4)	
Medication use	No in the last month	2 (3)	0	2 (1)	0.50 **
	Less than once a week	13 (19)	17 (24)	30 (21)	
	Once or twice a week	31 (44)	27 (39)	58 (41)	
	Three or more times a week	24 (34)	26 (37)	50 (36)	
Daytime sleepiness/frequency of problems	No problems	0	0	0	>0.99 **
	1–2	3 (4.3)	4 (5.7)	7 (5)	
	3–4	0	0	0	
	5–6	67 (95.7)	66 (94.3)	133 (95)	

* χ^2^ test; ** Fisher’s exact test.

**Table 5:** Sleep parameters measured by sleep quality index (PSQI) in relation to type of shift work.

	Median (Interquartile Range)		Difference (95% Confidence Range)	*p* *
	Nurses Working Day Shifts	Nurses Working Rotating Shifts		
Subjective sleep quality score	0 (0–0)	0 (0–0)	0 (0 to 0)	0.27
Total sleep quality index (PSQI)	9 (8–11)	10 (8–12)	0 (−1 to 1)	0.55

* Mann–Whitney U test (Hodges–Lehmann median difference).

**Table 6:** Sleep parameters measured using a smartwatch in relation to type of shift work.

	Median (Interquartile Range)		Difference (95% Confidence Range)	*p* *
	Nurses Working Day Shifts	Nurses Working Rotating Shifts		
Total sleep time (hours)	6.4 (6.3–7.1)	5.5 (5.2–6.2)	−1 (−1.2 to −0.9)	<0.001
Sleep score	77 (75–80)	73 (68–76)	−5 (−7 to −3)	<0.001
Deep sleep (hours)	1.11 (1.01–1.22)	1.02 (0.52–1.14)	−0.1 (−0.22 to −0.02)	0.01
Light sleep (hours)	3.8 (3.3–4.3)	3.3 (3.1–3.6)	−0.5 (−0.85 to −0.18)	<0.001
REM phase (hours)	1.25 (1.07–1.41)	1.12 (0.59–1.28)	−0.15 (−0.28 to −0.05)	0.003
Step count	7295 (6254–9823)	10,662 (7992–12,321)	2556 (1516 to 3619)	<0.001

* Mann–Whitney U test (Hodges–Lehmann median difference).

**Table 7:** Sleep quality assessment in relation to type of shift work according to the smartwatch sleep score.

	Number (%) of Respondents		Total	*p* *
	Nurses Working Day Shifts	Nurses Working Rotating Shifts		
Poor sleep quality	0	6 (9)	6 (4)	<0.001
Moderate sleep quality	47 (67)	57 (81)	104 (74)	
Good sleep quality	23 (33)	7 (10)	30 (21)	

* Fisher’s exact test.

## Data Availability

Data will be made available upon request by emailing the first author.
